# Alteration of putaminal fractional anisotropy in Parkinson’s disease: a longitudinal diffusion kurtosis imaging study

**DOI:** 10.1007/s00234-017-1971-3

**Published:** 2018-01-24

**Authors:** Yulia Surova, Markus Nilsson, Björn Lampinen, Jimmy Lätt, Sara Hall, Håkan Widner, Danielle van Westen, Oskar Hansson

**Affiliations:** 1 Department of Clinical Sciences, Lund, Lund University, Lund, Sweden; 2 Department of Neurology Lund, Skåne University Hospital, 221 85 Lund, Sweden; 3 Center for Medical Imaging and Physiology, Skåne University Hospital, Lund, Sweden; 4 Lund University Bioimaging Center, Lund University, Lund, Sweden; 5 Department of Medical Radiation Physics, Lund University, Lund, Sweden; 6 Department of Clinical Sciences, Malmö, Lund University, Malmö, Sweden; 7 Memory Clinic, Skåne University Hospital, Lund, Sweden

**Keywords:** Parkinson’s disease, Diffusion kurtosis imaging, Tractography, Tract-based spatial statistics

## Abstract

**Purpose:**

In Parkinson’s disease (PD), pathological microstructural changes occur that may be detected using diffusion magnetic resonance imaging (dMRI). However, there are few longitudinal studies that explore the effect of disease progression on diffusion indices.

**Methods:**

We prospectively included 76 patients with PD and 38 healthy controls (HC), all of whom underwent diffusion kurtosis imaging (DKI) as part of the prospective Swedish BioFINDER study at baseline and 2 years later. Annualized rates of change in DKI parameters, including fractional anisotropy (FA), mean diffusivity (MD), and mean kurtosis (MK), were estimated in the gray matter (GM) by placing regions of interest (ROIs) in the basal ganglia and the thalamus, and in the white matter (WM) by tract-based spatial statistics (TBSS) analysis.

**Results:**

When adjusting for potential confounding factors (age, gender, baseline-follow-up interval, and software upgrade of MRI scanner), only a decrease in FA in the putamen of PD patients (*β* = − 0.248, *P* < .01) over 2 years was significantly different from the changes observed in HC over the same time period. This 2-year decrease in FA in the putamen in PD correlated with higher l-dopa equivalent dose at baseline (Spearman’s rho = .399, *P* < .0001).

**Conclusion:**

The study indicates that in PD microstructural changes in the putamen occur selectively over a 2-year period and can be detected with DKI.

**Electronic supplementary material:**

The online version of this article (10.1007/s00234-017-1971-3) contains supplementary material, which is available to authorized users.

## Introduction

Parkinson’s disease (PD) is an incurable disorder that affects about ten million people worldwide [13]. There is a need for objective methods to track the disease progression over time in the symptomatic phase of patients with PD, to be used when evaluating novel disease-modifying therapies. Although longitudinal studies using positron emission tomography (PET) have been conducted in PD [29], radiation risk, cost, and infrastructural support limit their clinical utility. Diffusion magnetic resonance imaging (dMRI) is a non-invasive and widely accessible imaging modality, which makes it suited for longitudinal studies. A few studies have indicated that diffusion tensor imaging (DTI) might have a potential to track the disease progression in patients with PD [24, 28, 46]. Diffusion kurtosis imaging (DKI) is an extension of DTI [15] that has been suggested to be more sensitive in detecting and differentiating alterations of tissue microstructure [3, 47] and in PD patients [17, 45]. However, DKI tends to show inconsistent results in brain regions involved in the basal ganglia circuit in PD. Cross-sectional studies in patients with PD have reported increased mean diffusional kurtosis (MK) in basal ganglia, thalamus, and sensorimotor cortex [20, 43, 45], lower MK in white matter (WM) regions [17, 18], or no differences in MK in WM and deep gray matter in PD [37]. DKI has also been suggested as being sensitive to alpha-synuclein accumulation in transgenetic mice [21]. However, there are to our knowledge no longitudinal DKI studies available in PD.

To investigate the link between diffusional changes and PD progression and to distinguish between the concomitant effects of normal aging and disease evolution over time, a longitudinal approach involving both PD patients and normal subjects is the most appropriate study design. Investigation of the longitudinal cerebral alterations occurring in PD ideally includes baseline and follow-up data [23] using the same MRI scanner.

Thus, a 2-year prospective and longitudinal study was conducted on a cohort of PD patients and controls. A follow-up period of approximately 2 years is often used in trials evaluating novel disease-modifying therapies in neurodegenerative disorders such as PD. Effects of disease progression and normal aging on DKI measures in the basal ganglia, the thalamus, and cerebral WM tracts were investigated. Our specific aim was to determine whether there are specific DKI changes over time in PD patients when compared to healthy elderly over a 2-year time period.

## Materials and methods

### Ethics statement

This study was approved by the Ethics Committee at Lund University and performed in accordance with the Helsinki Declaration. All participants gave written informed consent prior to participation.

### Participants

In this case-control study, participants were recruited from the Neurology Clinic at Skåne University Hospital, Sweden, as part of the prospective and longitudinal Swedish BioFINDER study (www.biofinder.se) [12]. As of November 2016, 229 individuals had been included in the Parkinson sub-study of BioFINDER, of which 67 did not perform DKI at baseline, 39 did not perform DKI during follow-up, and 8 cases did not pass QC (Online Resource 1). Consequently, we included 76 patients with PD and 38 healthy controls. For the present work, 76 subjects were included with a clinical diagnosis of probable PD. The diagnosis was made by neurologists trained in movement disorder diagnostics according to the National Institute of Neurological Disorders and Stroke (NINDS) criteria of PD [10]. Neurologically healthy controls (HC), who did not have any objective cognitive or parkinsonian symptoms, were also recruited (*n* = 38). All 114 subjects had clinical assessments and were scanned on two occasions on average 25.3 months apart, with a standard deviation of 4.3 months. Motor function and disease stage were evaluated using, e.g., Unified Parkinson’s disease rating scale motor part (UPDRS-III) [7] and Hoehn and Yahr staging scale (H&Y) [14]. The total score from the motor section of the UPDRS III was broken down into subscales for bradykinesia, rigidity, tremor, gait posture [32]. Cognitive assessments were conducted by trained physicians using a Mini Mental State Examinations (MMSE) [9]. To ensure standardization, assessments were conducted during patients “on” medication state, or fully responding to their PD medications (in the “on” state). The daily l-dopa equivalent dose (LEDD) was calculated (Table 1) [39]. At the time of testing, none of the patients exhibited any dyskinesia, dystonia, or other signs of involuntary movement.

**Table 1:** Subject characteristic

	Healthy controls (*n* = 38)		Parkinson’s patients (*n* = 76)				
	Baseline	Year 2	Baseline	Year 2	Group effect	Time effect	Interaction
Age, years	66.4 (8.1)	–	65.0 (10.8)	–	.801	–	–
Sex (male/female)	16:22	–	52:24	–	.009	–	–
dMRI interval, month	–	25.9 (2.3)	–	25.3 (4.3)	.414	–	–
Disease duration, years	–	–	5.5 (3.6)	7.5 (3.6)	–	< .001	–
Bradykinesia	0 (.0)	0 (.0)	.7 (0.7)	.8 (.9)	< .001	.360	.360
Rigidity	.5 (.9)	.1 (.4)	1.8 (2.5)	1.9 (2.5)	< .001	.383	.293
Tremor	.0 (.3)	.1 (.4)	3.8 (3.3)	3.1 (3.1)	< .001	.256	.221
Gait/posture	.0 (.2)	.0 (.2)	.6 (1.0)	.8 (1.1)	< .001	.313	.313
Hoehn and Yahr stage	–	–	1.8 (.6)	2.0 (.9)	–	.237	–
UPDRS motor, score	1.4 (2.5)	1.7 (2.2)	12.5 (8.6)	14.0 (10.3)	< .001	.215	.347
MMSE, score	28.2 (1.6)	29.0 (1.2)	29.2 (4.5)	29.3 (4.7)	.390	.006	.024
LEDD_TOTAL_, mg	–	–	527.4 (374.9)	705.8 (350.6)	–	< .001	–

*UPDRS-III* unified Parkinson’s disease rating scale motor part, *MMSE* Mini Mental State Examination test, *LEDD* daily l-dopa equivalent dose

### MRI data acquisition

Imaging was performed on a 3 T Siemens Magnetom Skyra MR scanner equipped with a 20 channel head coil. The dMRI protocol comprised 99 DWI volumes, where the choice of b-values and encoding directions was inspired by Poot et al. [31]. In total, three volumes were acquired with *b* = 0 s/mm^2^, while the remaining 96 volumes were acquired using b-values of 250, 500, 1000, and 2750 s/mm^2^, distributed over 6, 6, 20, and 64 directions, respectively. A single-shot spin-echo with mono-polar diffusion encoding and EPI read-out was used for the acquisition with the following settings: voxel size = 2.3 × 2.3 × 2.3 mm^3^, FOV = 294 × 294 × 120 mm^3^, iPAT = 2, and partial Fourier factor = 6/8. The imaging volume comprised 52 contiguous axial slices adjusted to include the whole cerebrum. Total acquisition time was approximately 14 min. The study was initiated with the scanner on software version Syngo MR D11, but was later upgraded to version D13, and later again to E11. The upgrades resulted in slight changes of the repetition time (TR) and echo time (TE). For the D11, D13, and E11 versions, TR was set to 7500, 8100, and 8100 ms, respectively, while TE was set to 103, 103, and 104 ms, respectively. Some participants were scanned on baseline with D11 and on follow-up with D13 (28 HC and 41 PD), some made both baseline and follow-up scans on D13 (10 HC and 12 PD), while others made baseline scans on D13 and follow-up on E11 (23 PD). This was corrected for in the analysis, as described under the “analysis” section.

### Post-processing

Motion and eddy-current distortions were corrected using volume registration to extrapolated references, which is a method particularly well suited for high b-value data acquired in elderly subjects with atrophy [26]. In this procedure, the diffusion-weighted images were modulated with the Jacobian determinant of the transformation matrix [16]. In order to mitigate the potential effects of Gibbs ringing artifacts, image volumes were smoothed using an isotropic 3D Gaussian kernel with a full-width at half maximum of 2.3 mm [19, 30, 41]. Smoothing with a kernel of this size has little effect on sensitivity and specificity [40] and is thus not expected to significantly influence the parameter precision. DKI analysis was performed to obtain maps of fractional anisotropy (FA), MK, and mean diffusivity (MD), using in-house developed software which fitted the diffusion and kurtosis tensors by non-linear optimization as in [22]. The fitting only allowed positive values of the diffusion tensor eigenvalues. In a small number of voxels where the kurtosis was below zero, the fitting was repeated after additional smoothing was performed.

Longitudinal change was assessed in maps produced by coregistering by registering the data from the two time points. To reduce measurement bias, baseline and follow-up FA volumes were both registered to a subject-specific time-averaged template, after which the transform from the FA registration was applied to other contrasts. The template was created by, for each subject, computing and applying the transform that took the baseline volume half-way to the follow-up volume, and vice versa for the follow-up volume, and averaging the two. These registration steps were performed using non-linear registration with the FNIRT tool from FSL. All subsequent analysis was based on data in this subject-specific time-averaged space.

### Analysis

#### ROI based analysis of gray matter

Our a-priori hypothesis defined the caudate, putamen, globus pallidum, thalamus, substantia nigra, and red nucleus as regions of interest for PD follow-up. Our ROI-selection was based on a-priori hypothesis concerning four brain circuits or regions. The first was the cortico-basal ganglia circuit, which consists of the striatum (caudate nucleus and putamen), globus pallidus, and thalamus [33]. The second and third regions included the midbrain and the pons, which have also been implicated in PD [4, 11]. The fourth region comprised the red nucleus, which according to Braak has not been implicated in PD [4] and thus served as a reference region.” DKI values were obtained by region of interest (ROI) analysis. One experienced rater drew all ROIs manually according to Surova et al. [37] in the subject-specific time-average data. The same ROIs could thus be applied to both time points. The rater was blinded to the group (HC or patient). Separate ROIs were drawn in the left and the right hemispheres. Because of the presence of bilateral disease in all patients, laterality was not considered in the current study. Values from left and right hemispheres were thus averaged to obtain the final value for analysis. Intra-rater reliability for the ROI placement procedures were assessed on 23 randomly chosen participants using the FA, MD, and MK of the left-side ROIs as a quantitative measure and the mean interclass correlation coefficients for each ROI are presented in (Online Resource 2).

#### Tract-based spatial statistics analysis of white matter

We assessed differences in two-year changes in major WM tracts between PD and HC using tract-based spatial statistics (TBSS) (v 1.03), part of the FRMIB Software Library (FSL), which is a registration tool for improved voxel-wise comparisons between multiple subjects. The TBSS procedure involved registration of 2-year difference maps of FA, MK, and MD onto the 1 mm^3^ FMRIB58 FA template in MNI152 standard space, using the linear and non-linear registration tools FLIRT and FNIRT [1]. Before registration, the diffusion maps were masked with the FSL Brain Extraction Tool (BET) [35]. The normalized maps were then skeletonized by projection onto the FMRIB58 template skeleton. The skeletonized maps were subjected to voxel-wise comparison between PD and HC using FSL Randomize with 7500 permutations [44]. The procedure corrected for multiple comparisons using threshold-free cluster enhancement [36] and included age, gender, and software upgrade of the MRI scanner as covariates. TBSS analyses were done blinded for diagnosis.

### Analysis of white matter hyperintensities

Analysis of white matter hyperintensities (WMH) was rated according to the scales of Fazekas [8] and Wahlund [42].

### Statistical analysis

Statistical analysis of ROI data was performed with SPSS Statistics 20 for Windows (IBM Corporation, Somers, NY, USA). Demographic and clinical differences between groups were analyzed with either repeated measures ANOVA or Pearson’s chi-squared test. Correlations between diffusion parameters and clinical scores were tested for using the linear correlation coefficient (R2 Linear) and Spearman’s rho (R_s_). The change over time in the mean values (FA, MK, and MD) of the caudate nucleus, putamen, pallidum, thalamus, substantia nigra, and red nucleus were compared between the diagnostic groups using ANCOVA with age, gender, baseline follow-up interval, and software upgrade of MRI scanner included as covariates. Study participants who were scanned with the same scanner software version in baseline and follow-up was coded as “0,” while those were scanned with different software versions were coded as “1.” Significance threshold was set to 0.05. Multiple comparison correction was not applied to the reported *p* values. The TBSS analysis, however, inherently corrects for multiple comparisons through the threshold-free cluster enhancement procedure, as now mentioned above.

## Results

### Demographic and clinical

Table 1 shows the demographic and clinical characteristics of HC and patients with PD at baseline and after 2 years. There was no group difference in age but there was a significant difference in gender distribution (Pearson’s c^2^ = 7.2, *P* = .009), with a higher proportion of men in the PD group.

The PD group had higher UPDRS III total and sub-scores (*P* < .001) compared with HC. An effect of time was found for the MMSE (*P* = .006), due to an increase in the HC group over the 2 years, and for LEDD in the PD group (*P* < .001), amounting to a daily LEDD increase by approximately 35% (180 mg). There were no other significant changes in any other clinical or demographic data over time (Table 1).

### ROI based analysis of deep GM

**Fig. 1:**
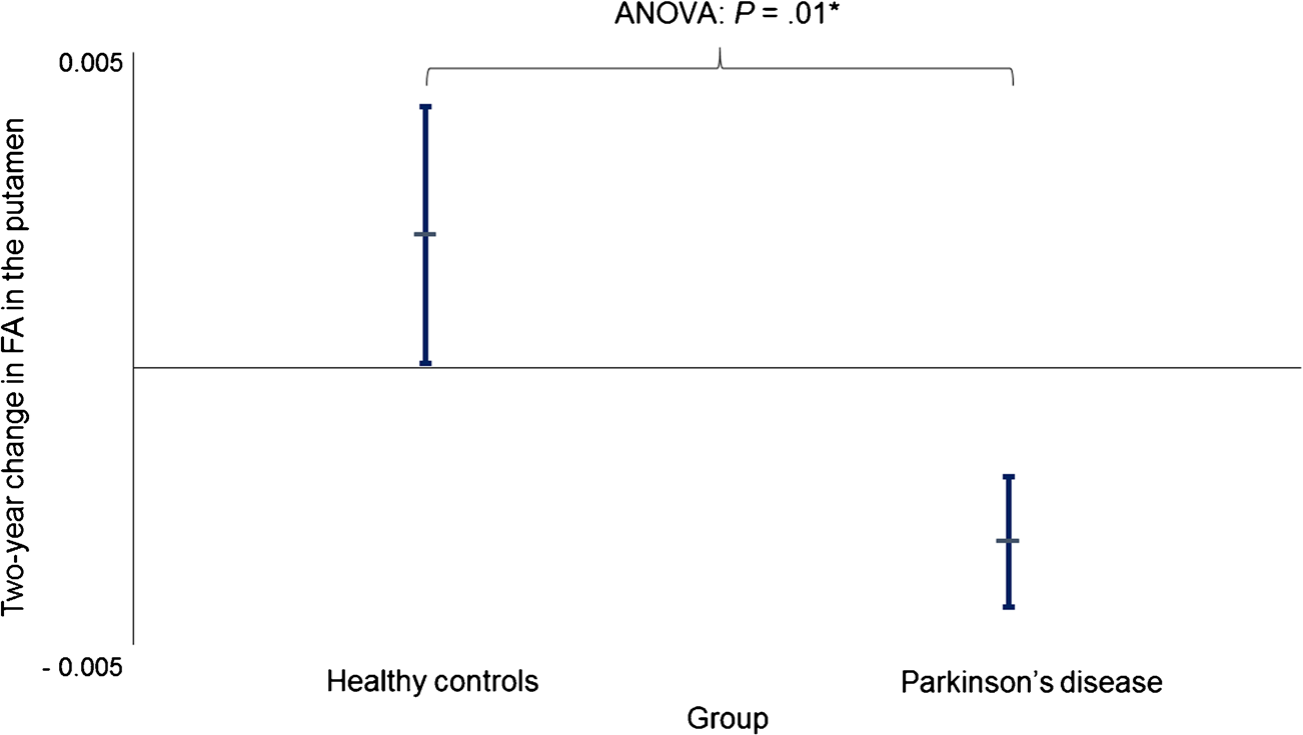
Two-year change of fractional anisotropy (FA) in the putamen in patients with Parkinson’s disease (PD) and healthy controls (HC). Differences in absolute values of FA (values from 2-year MRI minus values from baseline) in the putamen between patients with PD and HCs were analyzed using ANCOVA A modest significant difference was found between PD and HC (*β* = − 0.248, *P* = 0.01). Lines extending vertically indicate standard error of mean. Horizontal lines that intersect the vertical lines are means

Changes in DKI parameters were observed in both PD patients and controls over the 2-year period. Specifically, reductions in MD and increases in MK and FA were found in many brain regions (Online Resource 3). However, when comparing the changes in dMRI parameters in PD over 2 years to the changes observed in controls over the same time period, we only found a decrease in FA in the putamen in PD (ANCOVA, *β* = − .248, *P* = .001). Figure 1 illustrates FA changes in the putamen for PD and HC. There were no other significant longitudinal changes observed in PD when compared to controls, including in the white matter (Online Resource 3).

### Tract-based spatial statistics analysis of white matter

There were no significant longitudinal changes in either MD, FA, or MK in the WM observed in PD patients compared to controls.

### Correlation between clinical and DKI changes

**Fig. 2:**
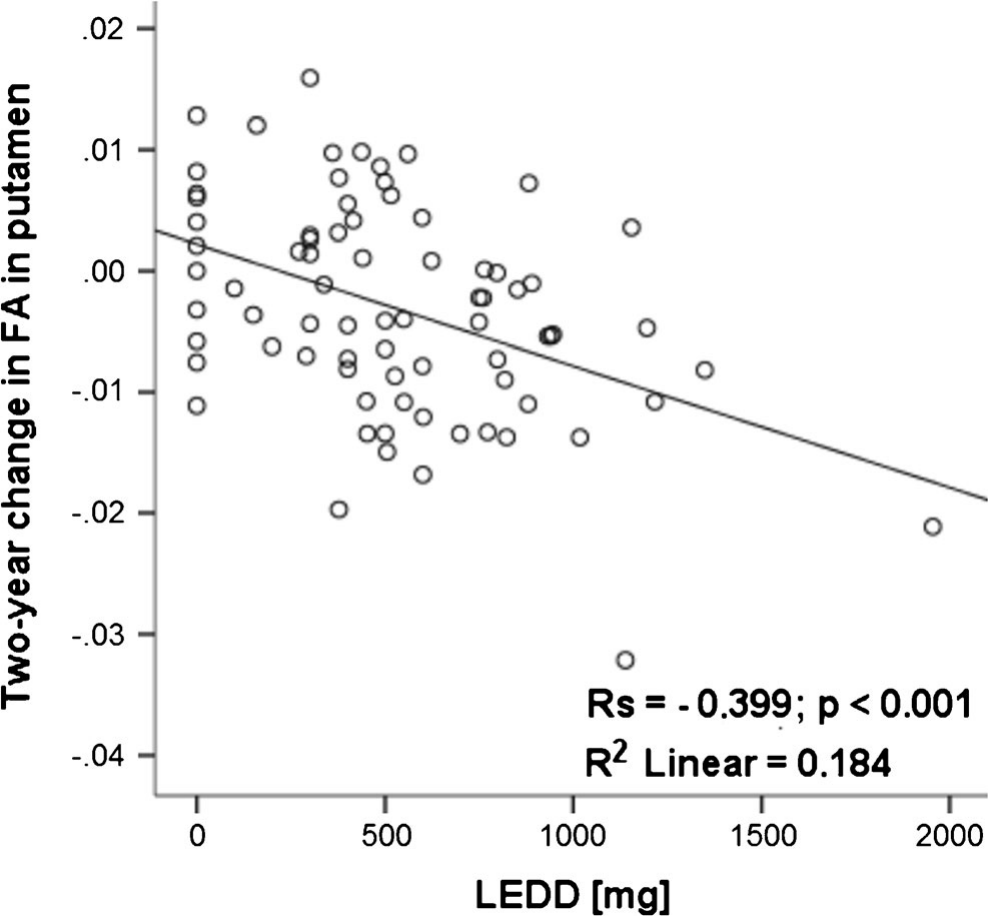
Correlation between 2-year change in fractional anisotropy (FA) in the putamen with the daily l-dopa equivalent dose (LEDD) at baseline. A moderate positive correlation was found between 2-year change in FA in the putamen and LEDD at baseline in PD. R2 linear, linear correlation coefficient; Rs, Spearman’s rho

Two-year change in FA in the putamen in PD patients correlated with LEDD at baseline (R2 linear = − 0.184; *R*_s_ = − 0.399, *P* < .0001) (Fig. 2) and LEDD at follow-up (data not shown). No correlations were found between DKI parameters and UPDRS III, H&Y, and MMSE. Also no correlations were found between change in DKI parameters versus change in UPDRS III, H&Y, and MMSE.

### Analysis of WMH

The amount of WMHs did not show any statistically significant difference between controls and patients with PD (data not shown).

## Discussion

We investigated disease-specific structural changes in deep GM and WM in patients with PD over a two-year period. In this cohort with 76 patients with PD and 38 controls, we found a selective reduction in FA in the putamen in PD that correlated with increased LEDD at baseline and follow-up. As the increased LEDD at follow-up is most probably the marker for disease progression, it might be that the decreased FA in the putamen at follow-up has some clinical relevance, especially if this result is confirmed by other studies. This result can be related to a recent longitudinal study that found an increased MD over 6 years in the anterior putamen in patients with PD [6] that correlated with UPDRS scoring [6]. Chan et al did not have controls [6], however, therefore their results may be affected by normal aging and system update effects such as those demonstrated here. Finally, our study did not show any significant diffusion changes in white matter over time in PD when compared to the changes observed in controls, which is in agreement with a previous study [34].

The observed putaminal FA changes may be related to the progressive loss of dopaminergic nerve terminals in the putamen that occurs in PD patients [5, 27]. Axonal damage or demyelination, with disruption of the axonal membrane and myelin sheath, causes a reduction in the water diffusivity restriction that results in decreased FA indices [2, 25, 37].

Since diffusion changes in PD patients involve both aging and the specific disease evolution, longitudinal follow-up including age-matched controls is warranted to take both factors into account, as done in the present study. Since no significant age-related putaminal FA changes were observed in the control group, it is likely that the disease itself is the main explanation.

The current cohort was similarly matched to controls as in the recent longitudinal DTI reports in PD patients. However, the current study deviates from previous longitudinal reports at several points [6, 23, 24, 28, 34, 46]. The PD patients in the current cohort had longer disease duration, compared to some [24, 46], but shorter compared to other previous studies [6, 23, 34]. The time between dMRI scans was longer, compared to in Ofori et al. [28], Loane et al. [24] and Zhang et al. [46]. Benefits of the current study include using the DKI sequence, which can assess both classic diffusion measurements (FA and MD) as well as MK. Our study is the first longitudinal DKI study. This is the reason that our study benefits from increased power via its longitudinal design. Furthermore, compared to other longitudinal DTI studies, the patients in the current study did not refrain from antiparkinsonian medication prior to scanning; therefore, we cannot objectively compare the different PD cohorts regarding disease severity. The most notable difference in findings is the lack of diffusion changes in the substantia nigra in the current report, which stands in contrast to previous results [23, 24, 46]. Zhang et al. [46] demonstrated an FA reduction in the substantia nigra of 3.6 ± 1.4%/year from baseline, and increased radial and axial diffusivity in the thalamus of 8.0 ± 2.9%/year and 4.0 ± 1.5%/year, respectively. Such discrepancies were possibly caused by differences in dMRI techniques (DTI in Zhang et al. and DKI in our study). Another possible reason could be that the study of Zhang et al. was multicenter (16 US sites, 5 European, 1 Australian), which can influence the interplay between selected image acquisition parameters and factors including signal-to-noise ratio, image resolution, image distortion and thus, the results derived.

Our study has a number of limitations. First, because the PD diagnoses were not histopathologically confirmed, there is a possibility of misdiagnosis. However, the validity of the PD diagnosis is strengthened by the observation that, after being followed for 25 months, all patients continued to respond satisfactorily to antiparkinsonian therapy and remained free of signs that are atypical Parkinsonism. Another limitation is that the ROIs on gray matter were drawn manually, which could lead to larger variability and bias. However, the interclass correlation coefficients indicated a low variability from this source when it came to putamen (0.81–0.91). Finally, since the study is longitudinal in nature, there is always a risk that those patients with a more severe disease progression drop out of the study, which might cause a selection bias of those still remaining in the study.

## Conclusion

Our longitudinal 2-year study of a relatively large cohort of PD patients provides evidence that DKI of the putamen can be used to detect disease progression in symptomatic PD patients. If this finding is replicated in other prospective and longitudinal DKI studies of PD patients and age-matched controls, DKI of putamen might be considered as a secondary outcome measure in clinical trials evaluating novel disease-modifying therapies. However, future studies also need to compare the accuracy of diffusion imaging of putamen with volumetric measures of the basal ganglia when it comes to tracking disease progression.

## Electronic Supplementary Material

Online Resource 1 (GIF 159 kb)

High Resolution Image (TIFF 159 kb)

Online Resource 2 (DOCX 17 kb)

Online Resource 3 (DOCX 16 kb)
