# Supplementary figures and images for: Alteration of putaminal fractional anisotropy in Parkinson’s disease: a longitudinal diffusion kurtosis imaging study
Source: Neuroradiology. 2018 Jan 24;60(3):247–54. doi: 10.1007/s00234-017-1971-3 (PMC5799343; doi:10.1007/s00234-017-1971-3)

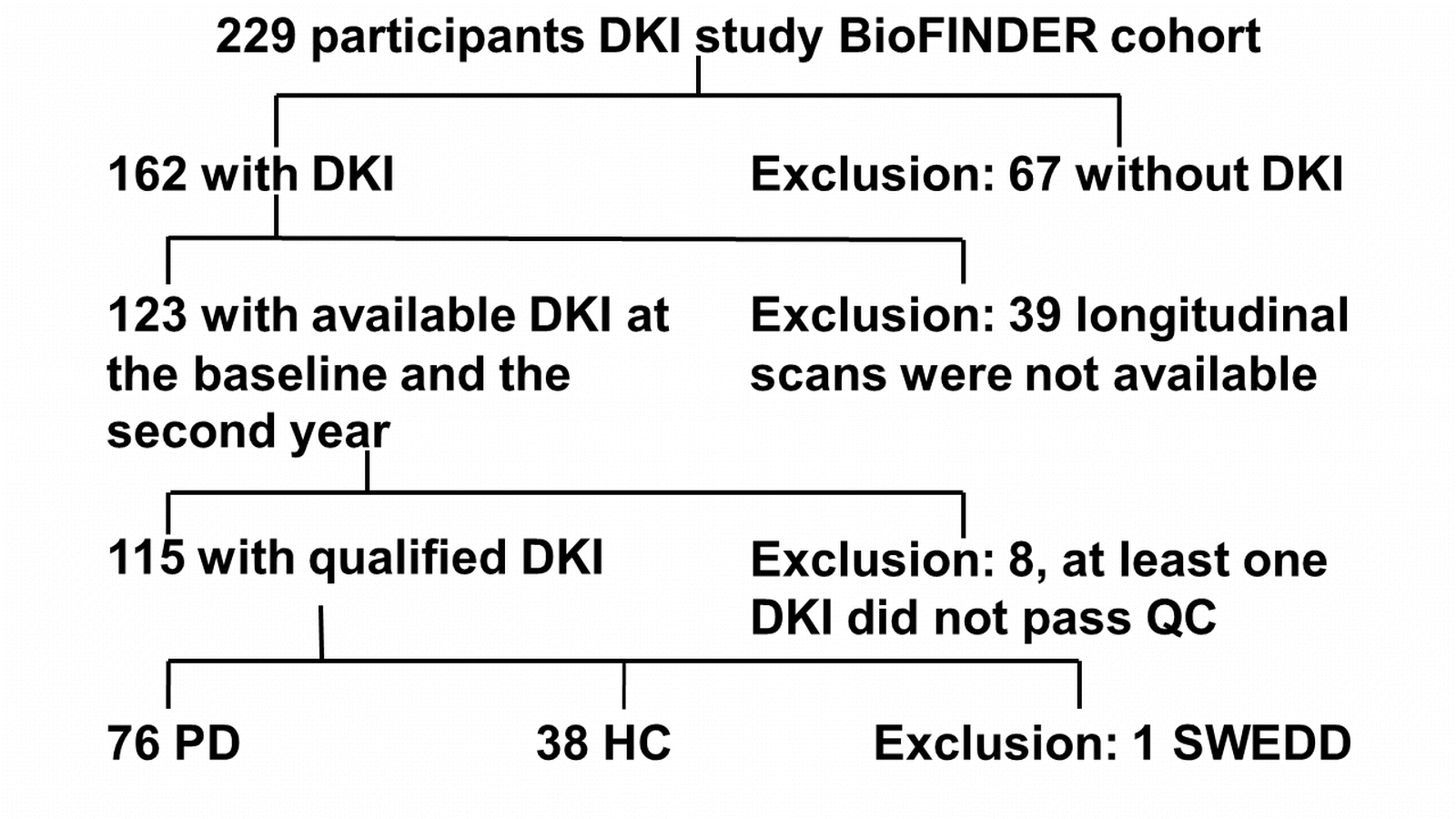

Supplement: Supplementary file 1 — (GIF 159 kb) [file 234_2017_1971_Fig3_ESM.gif]

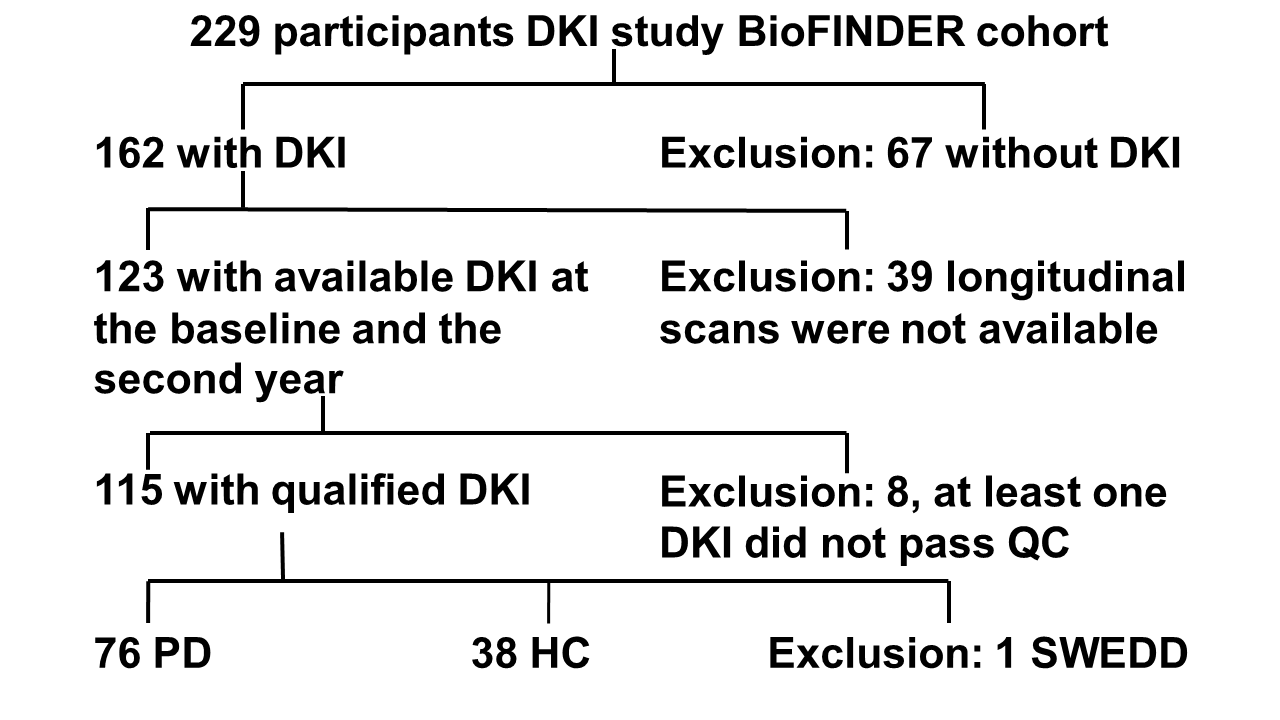

Supplement: Supplementary file 2 — High Resolution Image (TIFF 159 kb) [file 234_2017_1971_MOESM1_ESM.tif]
