# Supplementary material for: Alteration of putaminal fractional anisotropy in Parkinson’s disease: a longitudinal diffusion kurtosis imaging study
Source: Neuroradiology. 2018 Jan 24;60(3):247–54. doi: 10.1007/s00234-017-1971-3 (PMC5799343; doi:10.1007/s00234-017-1971-3)
Supplement: Supplementary file 3 — (DOCX 17 kb) [file 234_2017_1971_MOESM2_ESM.docx]

| **Table** Intraclass Correlation Coefficient as estimates of intra-rater reliability of ROI analysis | | | | | | | | |
| --- | --- | --- | --- | --- | --- | --- | --- | --- |
| Single Measures | Parameter | Intraclass  correlation | 95% Confidence Interval | | F Test With True Value 0 | | | |
|  |  |  | Lower Bound | Upper Bound | Value | df1 | df2 | Sig |
| Caudate head | FA | .632 | .311 | .825 | 4.841 | 22 | 22 | .000 |
| Putamen | FA | .808 | .597 | .914 | 9.041 | 22 | 22 | .000 |
| Thalamus | FA | .909 | .801 | .960 | 21.206 | 22 | 22 | .000 |
| Pallidum | FA | .560 | .213 | .784 | 3.629 | 22 | 22 | .002 |
| Red nucleus | FA | .314 | -.100 | .650 | 3.072 | 22 | 22 | .006 |
| Substantia nigra | FA | .593 | -.043 | .850 | 7.647 | 22 | 22 | .000 |
| Caudate head | MD | .248 | -.121 | .577 | 1.758 | 22 | 22 | .097 |
| Putamen | MD | .833 | .622 | .928 | 12.692 | 22 | 22 | .000 |
| Thalamus | MD | .613 | .168 | .832 | 5.690 | 22 | 22 | .000 |
| Pallidum | MD | .654 | .338 | .837 | 4.666 | 22 | 22 | .000 |
| Red nucleus | MD | .698 | -.014 | .902 | 12.294 | 22 | 22 | .000 |
| Substantia nigra | MD | .753 | .141 | .916 | 12.977 | 22 | 22 | .000 |
| Caudate head | MK | .748 | .495 | .885 | 6.805 | 22 | 22 | .000 |
| Putamen | MK | .911 | .803 | .961 | 20.807 | 22 | 22 | .000 |
| Thalamus | MK | .967 | .866 | .989 | 89.018 | 22 | 22 | .000 |
| Pallidum | MK | .933 | .592 | .980 | 52.954 | 22 | 22 | .000 |
| Red nucleus | MK | .935 | .730 | .978 | 45.439 | 22 | 22 | .000 |
| Substantia nigra | MK | .895 | .605 | .953 | 27.650 | 22 | 22 | .000 |

The reliability of the ROI-placement procedures was calculated using the intraclass correlations coefficient (ICC) estimates and their 95% confident intervals were calculated based on a mean-rating (k = 1), single measures, absolute-agreement and 2-way mixed effects model (Koo and Li 2016).

FA, fractional anisotropy; MD, mean diffusivity [10^-9 m^2/s]; MK, mean kurtosis. Values less than 0.5 are indicative of poor reliability, values between 0.5 and 0.75 indicate moderate reliability, values between 0.75 and 0.9 indicate good reliability, and values greater than 0.90 indicate excellent reliability.
