# Supplementary material for: Alteration of putaminal fractional anisotropy in Parkinson’s disease: a longitudinal diffusion kurtosis imaging study
Source: Neuroradiology. 2018 Jan 24;60(3):247–54. doi: 10.1007/s00234-017-1971-3 (PMC5799343; doi:10.1007/s00234-017-1971-3)
Supplement: Supplementary file 4 — (DOCX 16 kb) [file 234_2017_1971_MOESM3_ESM.docx]

| **Table** DKI markers at baseline and at two years | | | | | |
| --- | --- | --- | --- | --- | --- |
|  |  | Healphy controls (n = 38) | | Parkinson’s patients (n = 76) | |
|  |  | Baseline | Year 2 | Baseline | Year 2 |
| Caudate head | FA | .203 (.025) | .202 (.029) | .200 (.024) | .196 (.028) ***** |
| Putamen | FA | .186 (.026) | .188 (.03) | .179 (.022) | **.176 (.024)*** |
| Thalamus | FA | .334 (.02) | .338 (.02) ***** | .332 (.02) | .335 (.02) ***** |
| Pallidum | FA | .276 (.037) | .282 (.041) ***** | .274 (.034) | .278 (.03) ***** |
| Red nucleus | FA | .481 (.048) | .493 (.052) ***** | .470 (.054) | .478 (.058) ***** |
| Substantia nigra | FA | .468 (.05) | .482 (.064) ***** | .463 (.051) | .467 (.06) |
| Caudate head | MD | .745 (.082) | .716 (.075) ***** | .786 (.111) | .762 (.101) ***** |
| Putamen | MD | .781 (.086) | .756 (.088) ***** | .825 (.118) | .804 (.125) ***** |
| Thalamus | MD | .789 (.045) | .766 (.048) ***** | .816 (.055) | .795 (.058) ***** |
| Pallidum | MD | .702 (.148) | .671 (.152) ***** | .746 (.116) | .724 (.118) ***** |
| Red nucleus | MD | .583 (.069) | .551 (.077) ***** | .610 (.087) | .592 (.089) ***** |
| Substantia nigra | MD | .629 (.111) | .599 (.118) ***** | .672 (.106) | .650 (.117) ***** |
| Caudate head | MK | .948 (.221) | .948 (.239) | .888 (.117) | .877 (.130) |
| Putamen | MK | 1.238 (0.228) | 1.239 (.238) | 1.138 (.129) | 1.134 (.138) |
| Thalamus | MK | 1.181 (0.095) | 1.185 (.098) | 1.136 (.087) | 1.139 (.107) |
| Pallidum | MK | 1.973 (0.379) | 2.042 (.401) ***** | 1.806 (.277) | 1.826 (.294) |
| Red nucleus | MK | 2.076 (0.316) | 2.122 (.339) ***** | 1.981 (.355) | 2.006 (.384) |
| Substantia nigra | MK | 2.226 (0.474) | 2.289 (.509) ***** | 2.048 (.386) | 2.090 (.43) ***** |

FA, factional anisotropy; MD, mean diffusivity; MK, mean kurtosis. The data are presented in FA, MD [10^-9 m^2/s] and MK as the mean (standard deviation). *Significant from baseline. Bold indicates when change in Parkinson’s patients different from change in controls over 2 years.
